# Geometry-Constrained Learning-Based Visual Servoing with Projective Homography-Derived Error Vector

**DOI:** 10.3390/s25082514

**Published:** 2025-04-16

**Authors:** Yueyuan Zhang, Arpan Ghosh, Yechan An, Kyeongjin Joo, SangMin Kim, Taeyong Kuc

**Affiliations:** Department of Electrical and Computer Engineering, College of Information and Communication Engineering, Sungkyunkwan University, Suwon 16419, Republic of Korea; yueyuan1696@skku.edu (Y.Z.); ghosharpan@skku.edu (A.G.); ycan@skku.edu (Y.A.); jookj96@skku.edu (K.J.); kimsang.m@g.skku.edu (S.K.)

**Keywords:** visual servoing, eye-in-hand configuration, geometry constraints, cerebellar model articulation controller

## Abstract

We propose a novel geometry-constrained learning-based method for camera-in-hand visual servoing systems that eliminates the need for camera intrinsic parameters, depth information, and the robot’s kinematic model. Our method uses a cerebellar model articulation controller (CMAC) to execute online Jacobian estimation within the control framework. Specifically, we introduce a fixed-dimension, uniform-magnitude error function based on the projective homography matrix. The fixed-dimension error function ensures a constant Jacobian size regardless of the number of feature points, thereby reducing computational complexity. By not relying on individual feature points, the approach maintains robustness even when some features are occluded. The uniform magnitude of the error vector elements simplifies neural network input normalization, thereby enhancing online training efficiency. Furthermore, we incorporate geometric constraints between feature points (such as collinearity preservation) into the network update process, ensuring that model predictions conform to the fundamental principles of projective geometry and eliminating physically impossible control outputs. Experimental and simulation results demonstrate that our approach achieves superior robustness and faster learning rates compared to other model-free image-based visual servoing methods.

## 1. Introduction

Visual servoing utilizes visual feedback to refine robot motion strategies, thereby significantly enhancing adaptability and intelligence. The visual system can be mounted on the end effector of the robot (eye-in-hand configuration), or positioned near the robot (hand-to-eye configuration) [1]. This study mainly focuses on the eye-in-hand configuration.

Visual servoing is generally divided into two main approaches: position-based visual servoing (PBVS) and image-based visual servoing (IBVS) [2,3,4]. PBVS operates in Cartesian space, enabling global convergence by directly associating image features with the pose of the target in 3D space. However, the PBVS is sensitive to camera calibration errors, inaccuracies in the kinematic model, and noise, often requiring precise calibration, making it challenging for non-experts. In contrast, IBVS defines its error signal in the image space, making it less sensitive to calibration errors that primarily affect convergence speed rather than accuracy. Nevertheless, IBVS is prone to local minima, and the appearance or disappearance of feature points during the control process can significantly disrupt the continuity of the control law [5]. An alternative approach is homography-based visual servoing (HBVS), which combines the advantages of PBVS and IBVS. HBVS is resilient to the partial occlusion of features and does not require prior information for 3D reconstruction. Studies [6,7,8,9] have decomposed the homography matrix to obtain the translation vector t and rotation matrix R, converting the image information into motion in the Cartesian space. However, decomposing the homography matrix during the control process increases computational demands and affects the real-time performance of the system. To circumvent this issue, one study [10] has proposed constructing a cost function directly from the elements of the homography matrix. However, this method often requires vanishing-point detection. To address these challenges, projective homography-based uncalibrated visual servoing (PHUVS) was introduced [11]. The PHUVS establishes the task function by estimating the projective homography using only image information within a certain range, eliminating the need for vanishing point detection. Moreover, it can produce Jacobian matrices of fixed dimensions, thereby reducing the computational complexity when inverting matrices.

Visual servoing methods typically rely on a deeper understanding of the Jacobian matrix. This matrix describes the mapping between the visual information and manipulator joint angular displacements. It is a local linear approximation of the nonlinear and highly coupled relationship between the visual and motion spaces, making it crucial for control algorithms. Traditional methods usually require accurate system kinematic models or precise estimations of the intrinsic camera parameters. These requirements significantly limit their applicability. To address the challenges of uncalibrated visual servoing, adaptive controllers have been proposed to estimate unknown parameters online [12,13,14]. However, these methods often require linear parameterization of the system modeling, which is complicated for nonlinear systems with multiple degrees of freedom. Consequently, model-free learning methods have been introduced to compensate for these uncertainties without the need for linear parameterization. These methods treat the estimation of a Jacobian matrix as a dynamic parameter identification problem. They employ recursive estimation techniques such as weighted recursive least squares, Broyden’s method, and dynamic Gauss–Newton algorithms [15,16]. Neural networks are renowned for their powerful nonlinear function approximation capabilities and have been widely applied in robotic systems [17,18,19,20,21,22,23]. However, their application in visual servoing systems is relatively limited. In [17], a method based on perceptron neural networks was proposed to learn the inverse mapping of an unknown interaction matrix; however, it required a large amount of data for offline training. Data-driven online mapping estimation methods have been designed [18,19]. In addition, ref. [20] proposed a data-driven IBVS method that addresses both the target tracking and physical constraints of the robot, such as joint velocity limits and FOV constraints. The study in [21] presented a robotic control system that uses a CMAC network with a Takagi–Sugeno fuzzy framework, capable of online learning joint velocities directly from image feature errors. Recent works have also explored deep learning-based visual servoing frameworks with improved robustness and noise tolerance. For instance, ref. [22] employs deep networks for 3D visual servoing without requiring precise calibration, while [23] introduces a noise-tolerant Jacobian estimation method using neural networks under pixel-level disturbances.

Traditional visual servoing systems often rely on handcrafted features such as SIFT or ORB, which can be sensitive to noise, lighting changes, and viewpoint variations. To improve robustness, learning-based methods like SuperPoint [24] and LightGlue [25] have been developed. These methods offer improved feature repeatability and matching accuracy under challenging conditions, making them promising complements to learning-based visual servoing frameworks.

Current visual servoing algorithms face two main challenges: (1) Existing model-free visual servoing approaches are predominantly based on IBVS. The loss of feature points can cause interruptions in the control process. However, due to changes in lighting and partial occlusion of feature points, it is difficult to ensure that every feature point is detected and accurately matched during control. Hence, IBVS is primarily designed for basic visual primitives, such as distinct points or edges. For objects with sparse or low-texture surfaces, feature matching errors become significantly higher, further degrading the reliability of the control process. Furthermore, the size of the image Jacobian matrix is proportional to the number of feature points, resulting in high computational costs when calculating the Jacobian pseudoinverse. When feature points are used as the inputs or outputs of a neural network, an increase in their number significantly expands the network’s dimensionality, thereby affecting training efficiency and computational complexity. This not only increases the number of network parameters but may also lead to optimization issues such as gradient vanishing or gradient explosion, ultimately impacting convergence speed and stability. (2) Current model-free visual servoing methods typically use neural networks or recursive algorithms to learn the Image Jacobian or map visual features to control commands. However, these approaches often ignore the geometric constraints among visual features, resulting in parameter updates that violate physical laws during training. Without explicitly incorporating these constraints, robots may execute unreasonable motions.

We develop a geometry-constrained learning-based control strategy based on PHUVS. The main contributions of this paper include:

1. Learning-Based Control Strategy: A novel control method is proposed for PHUVS, where the CMAC neural network is employed to estimate the Jacobian matrix online. This eliminates the need for traditional calibration and kinematic modeling.
2. Fixed-Dimension Visual Error Function via Homography: A new visual error function based on the projective homography matrix is designed. It maintains a fixed dimension and uniform magnitude across all components, thereby improving learning efficiency, reducing computational complexity, and offering robustness against feature point occlusion and detection errors.
3. Incorporation of Geometric Constraints in Learning: Fundamental geometric relationships among visual features (e.g., collinearity) are embedded into the CMAC network’s learning process, enhancing both accuracy and convergence speed.

The remainder of this paper is organized as follows: Section 2 provides an overview of the camera/robot model and the projective homography matrix, while clearly defining the control problem. Section 3 details the developed controller. Section 4 presents the stability analysis of the proposed control system, providing theoretical guarantees for the controller’s performance. In Section 5, we analyze the simulation and experimental results, to demonstrate the efficacy of proposed methodology. Section 6 presents the conclusions and highlights the contributions of the study.

## 2. Preliminary Knowledge

### 2.1. Kinematics Model

**Figure 1:**
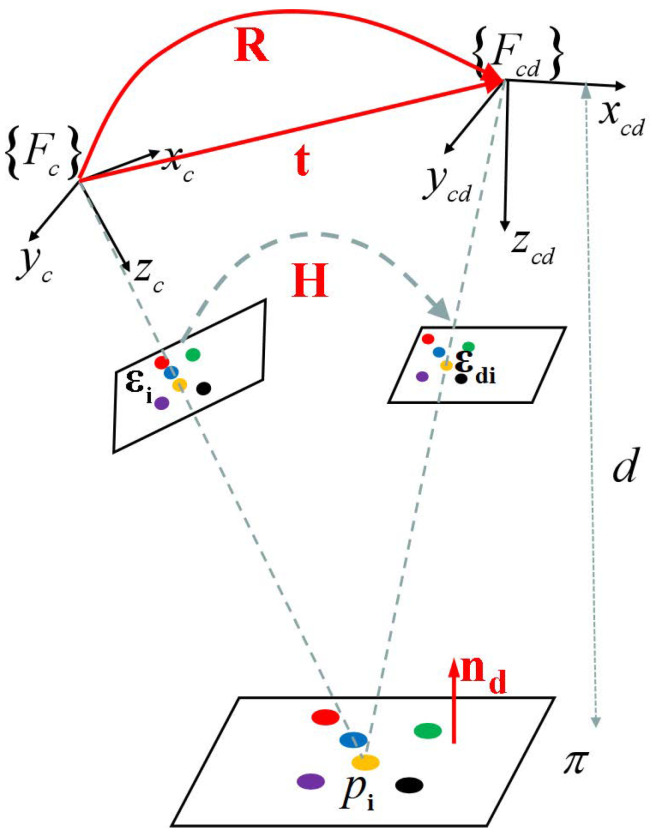
Camera model.

As shown as Figure 1, {F_c_} and {F_cd_} represent the current and desired camera frames, respectively. Consider $n_{d}$ as the normal vector of the plane π expressed in {F_cd_} with length $∥n_{d}∥\;=\frac{1}{d}$. Here, d is the distance between the plane and center of the projection at the reference pose. R denotes the orientation of {F_cd_} with respect to {F_c_}, and t denotes the translation vector from {F_c_} to {F_cd_} expressed in the coordinate frame {F_c_}.

Let $p_{i}$ be the point of the target in 3D space. The Euclidean coordinates expressed in {F_c_} and {F_cd_} are denoted:

(1) $$p_{i}^{c}=Hp_{i}^{\mathrm{cd}},$$

(2) $$H=R+tn_{d}^{T},$$

where H is the Euclidean homography matrix. This matrix describes how the 3D points lying on a planar surface are mapped between two Euclidean frames.

The projections of the 3D points into two image spaces are: ${\left[\begin{array}{cc} \varepsilon_{\mathrm{di}} & 1 \end{array}\right]}^{T}=K\frac{p_{i}^{\mathrm{cd}}}{Z_{di}}$ and ${\left[\begin{array}{cc} \varepsilon_{i} & 1 \end{array}\right]}^{T}=K\frac{p_{i}^{c}}{Z_{i}}$, where K is the camera intrinsic parameter matrix.

Subsequently, the relationship between $\varepsilon_{i}$ and $\varepsilon_{\mathrm{di}}$ will be:

(3) $$\left[\begin{array}{c} \varepsilon_{i}\\ 1 \end{array}\right]=\frac{Z_{di}}{Z_{i}}G\left[\begin{array}{c} \varepsilon_{\mathrm{di}}\\ 1 \end{array}\right],$$

(4) $$G=\mathrm{KH}K^{-1},$$

where G is the projective homography matrix. In the absence of depth information, we can compute the matrix G up to a scale factor using only four or more non-collinear matched points $\left\{\varepsilon_{i},\varepsilon_{\mathrm{di}}\right\},i\in \left\{1,2,3,4\cdots \right\}$:$\bar{G}=\beta G$.

$\bar{G}$ is an estimation of G and the parameter β∈R represents an arbitrary positive scale factor. It is crucial to note that this random ratio is independent of the depth ratio: $\frac{Z_{di}}{Z_{i}}$. Typically, the determinant of the $\bar{G}$ is normalized to 1: $det\left(\bar{G}\right)=1$ to eliminate the ambiguity introduced by the scale factor. In this case, the matrix $\bar{G}$ can be represented as: T$\bar{G}=\frac{G}{det\left(G\right)}$. Then, the relationship between the positions of feature points in the two images can be rewritten as follows:

(5) $$\left[\begin{array}{c} \varepsilon_{i}\\ 1 \end{array}\right]=\gamma_{i}\bar{G}\left[\begin{array}{c} \varepsilon_{\mathrm{di}}\\ 1 \end{array}\right],$$

(6) $$\gamma_{i}=\frac{1}{{\bar{g}}_{3,:}{\left(\left[\begin{array}{cc} \varepsilon_{\mathrm{di}}^{T} & 1 \end{array}\right]\right)}^{T}},$$

where ${\bar{g}}_{3,:}$ is the 3rd row of the matrix $\bar{G}$. The subscript i denotes the index of the feature point. In the subsequent text, we use subscript c to denote the object center, where $\varepsilon_{\mathrm{dc}}$ and $\varepsilon_{c}$ represent its desired and current positions in image space, respectively. The values of $\bar{G}$ and $\gamma_{i}$ can be estimated by m (m > 4) pairs of non-collinear matched feature points. When $\bar{G}$ tends to the identity matrix $I_{3\times 3}$, it indicates that the two images are perfectly aligned.

In a camera-in-hand visual servoing setup, the camera is mounted on the robot’s end-effector. By solving the kinematics, the position vector $r_{c}^{b}$ of the camera in the base frame is a function of the joint variables:

(7) $$r_{c}^{b}=f\left(q\right),$$

where $q\in R^{M}$ is the joint angle vector of the manipulator, the spatial velocity of the end-effector can be obtained as:

(8) $$v_{c}^{b}=J_{r}\left(q\right)\dot{q},J_{r}\in R^{6\times n_{q}},$$

where $n_{q}$ is the number of robot joints, $J_{r}\left(q\right)$ is the robot Jacobian, $\dot{q}$ is the joint velocities, and $v_{c}^{b}={\left[v_{x},v_{y},v_{z},\omega_{x},\omega_{y},\omega_{z}\right]}^{T}$ is end-effector spatial velocity, which includes the linear velocity *v* and angular velocity ω.

### 2.2. Problem Description and Motivation

Equation (8) describes the velocity mapping from the joint space to the camera motion in Cartesian space. To obtain the mapping from the joint space to the image space, we must construct an image Jacobian, which relates the camera’s velocities to the resulting changes in the image space.

In the methods of IBVS [12,13,14,15,16,17,18,19,20,21], the image error ${\tilde{\varepsilon }}_{i}=\varepsilon_{\mathrm{di}}-\varepsilon_{i}$ is directly used as the feedback signal. Its derivative and mapping to the joint space can be expressed as follows:

(9) $$\dot{\tilde{\varepsilon }}=J_{c_{IBVS}}J_{r}\dot{q}=J_{IBVS}\dot{q},\;J_{IBVS}\in R^{2P\times n_{q}}$$

where $\dot{\varepsilon }={\left[{\dot{\varepsilon }}_{1}^{T},{\dot{\varepsilon }}_{2}^{T},\cdots {\dot{\varepsilon }}_{P}^{T}\right]}^{T}\in R^{2P\times 1}$. P and $n_{q}$ represent the numbers of feature points and robot joints, respectively. The image Jacobian matrix contains information on the feature point positions and image depth: $J_{c_{IBVS}}=\left[J_{c_{IBVS}}^{1}\left(\varepsilon_{1},Z_{1}\right);J_{c_{IBVS}}^{2}\left(\varepsilon_{2},Z_{2}\right);\ldots J_{c_{IBVS}}^{N}\left(\varepsilon_{P},Z_{P}\right)\right]$.

Although depth-independent Jacobian matrices have been developed in some studies, several common challenges persist. First, the dimension of the Jacobian is proportional to the number of feature points, implying that as the number of feature points increases, the computational complexity also increases. Second, the task function typically minimizes the position errors of all feature points. When some feature points are occluded, they become unavailable. Changes in the number of feature points can lead to discontinuities in the task function, resulting in discontinuities in the control law.

In study [11], a task error based on the PHUVS was designed as follows:

(10) $$e_{\bar{g}}=\mathrm{re}shape\left(I-\bar{G},9,1\right)$$

The expression linking the derivative of $e_{\bar{g}}$ with the camera velocity is given as:

$${\dot{e}}_{\bar{g}}=J_{c_{PHUVS}}\left(e_{\bar{g}}\right)v_{c}^{b},$$

where the image Jacobian $J_{c_{PHUVS}}\left(e_{\bar{g}}\right)\in R^{9\times 6}$. Combining with Equation (8), the relationship between ${\dot{e}}_{\bar{g}}$ and $\dot{q}$ can be determined as:

(11) $${\dot{e}}_{\bar{g}}=J_{PHUVS}\left(e_{\bar{g}},q\right)\dot{q},\;J_{PHUVS}\left(e_{\bar{g}},q\right)\in R^{9\times n_{q}}$$

where $J_{PHUVS}\left(e_{\bar{g}},q\right)=J_{c_{PHUVS}}\left(e_{\bar{g}}\right)\cdot J_{r}\left(q\right)$ denotes the system Jacobian matrix.

The error function $e_{\bar{g}}$ in Equation (10) consists of nine elements, each representing a different type of geometric transformation, such as rotation, scaling, perspective, and translation. In conventional neural networks, normalization is challenging due to the uncertain range of each element. Similarly, in the CMAC network, the input quantization process becomes difficult as the elements vary significantly in their numerical scales and physical meanings, complicating the discretization into appropriate regions.

To address the above issues, we first reconstructed a fixed-dimension, uniform-magnitude visual error function. It is important to note that the error function we designed differs from the one presented in reference [11], specifically defined in Equation (10). In contrast, our error function consists of only 10 elements, each with consistent physical meanings and uniform magnitude, which simplifies the input quantization process for the neural network.

Despite the improved error function, a key limitation remains: existing model-free learning approaches update network weights without explicitly enforcing geometric constraints. This omission can lead to physically inconsistent mappings, such as collinear points deviating from collinearity after projection. To address this, we integrate geometric constraints into the weight update process. Specifically, we incorporate regularization terms into the loss function to preserve geometric consistency and employ projected gradient descent to ensure physically meaningful updates.

## 3. Controller Development

### 3.1. Novel Fixed-Dimension, Uniform-Magnitude Task Error Function

Assume that the camera captures an image at a reference position. The objective is to adjust the pose of the robot to ensure the current image aligns with the reference image, effectively bringing the camera back to its reference position.

**Figure 2:**
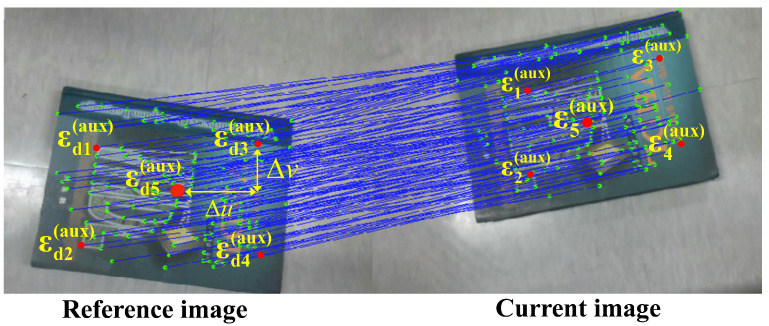
Image features.

This process involves the detecting and matching of image feature points. In Figure 2, the blue points represent the matched feature point pairs: $\left\{\varepsilon_{j},\varepsilon_{\mathrm{dj}}\right\},j=1,2\ldots P$, which are used to estimate the projective homography matrix $\bar{G}$. To ensure robust estimation of this transformation, we use enough feature points across both images. Having sufficient points provides stability in the homography estimation, as it reduces the sensitivity to noise or small mismatches at individual points. Robust estimation methods, such as random sample consensus (RANSAC), help filter out outliers and produce an accurate homography matrix $\bar{G}$.

To achieve a fixed-dimensional Jacobian, we introduce five fixed, non-collinear virtual reference feature points around the object center. The expected position of each virtual feature point in the reference image is defined as:

(12) $$\left[\begin{array}{c} \varepsilon_{d1}^{\left(\mathrm{aux}\right)}\\ \varepsilon_{d2}^{\left(\mathrm{aux}\right)}\\ \varepsilon_{d3}^{\left(\mathrm{aux}\right)}\\ \varepsilon_{d4}^{\left(\mathrm{aux}\right)}\\ \varepsilon_{d5}^{\left(\mathrm{aux}\right)} \end{array}\right]=\left[\begin{array}{c} \varepsilon_{\mathrm{dc}}^{\left(\mathrm{aux}\right)}+{\left[\begin{array}{cc} -\Delta u & \Delta v \end{array}\right]}^{T}\\ \varepsilon_{\mathrm{dc}}^{\left(\mathrm{aux}\right)}+{\left[\begin{array}{cc} -\Delta u & -\Delta v \end{array}\right]}^{T}\\ \varepsilon_{\mathrm{dc}}^{\left(\mathrm{aux}\right)}+{\left[\begin{array}{cc} \Delta u & \Delta v \end{array}\right]}^{T}\\ \varepsilon_{\mathrm{dc}}^{\left(\mathrm{aux}\right)}+{\left[\begin{array}{cc} \Delta u & -\Delta v \end{array}\right]}^{T}\\ \varepsilon_{\mathrm{dc}}^{\left(\mathrm{aux}\right)} \end{array}\right]$$

where $\varepsilon_{\mathrm{dc}}$ represents the center of the object in the reference image, and Δu and Δv are the displacements of the feature points in the u and v directions, respectively.

We define an error matrix as follows:

(13) $$E^{i}=I-\gamma_{i}^{\left(\mathrm{aux}\right)}\bar{G}$$

(14) $$\gamma_{i}^{\left(\mathrm{aux}\right)}=\frac{1}{{\bar{g}}_{3,:}{\left[\begin{array}{cc} \varepsilon_{\mathrm{di}}^{\left(\mathrm{aux}\right)T} & 1 \end{array}\right]}^{T}}$$

The actual position of the i-th virtual feature point in the current image is denoted as $\varepsilon_{i}^{\left(\mathrm{aux}\right)}$. Based on Equations (5), (6), (13) and (14), the position error can be rewritten as:

(15) $${\tilde{\varepsilon }}_{i}^{\left(\mathrm{aux}\right)}=\varepsilon_{\mathrm{di}}^{\left(\mathrm{aux}\right)}-\varepsilon_{i}^{\left(\mathrm{aux}\right)}=E_{1:2,:}^{i}{\left[\begin{array}{cc} \varepsilon_{\mathrm{di}}^{\left(\mathrm{aux}\right)T} & 1 \end{array}\right]}^{T}=e_{i},$$

where $E_{1:2,:}^{i}$ represents the first two rows of the matrix $E^{i}$.

Furthermore, $e_{i}$ is our designed error task function for the i-th feature. If the estimation $\bar{G}$ is precise, $e_{i}$ is equivalent to the feature point position error ${\tilde{\varepsilon }}_{i}^{\left(\mathrm{aux}\right)}$.

We combine the error vectors for the four virtual feature points into a single error task vector:

(16) $$e=\left[\begin{array}{c} e_{1}\\ e_{2}\\ \vdots \\ e_{5} \end{array}\right]=\left[\begin{array}{c} E_{1:2,:}^{1}\varepsilon_{d1}^{\left(\mathrm{aux}\right)}\\ E_{1:2,:}^{2}\varepsilon_{d2}^{\left(\mathrm{aux}\right)}\\ \vdots \\ E_{1:2,:}^{5}\varepsilon_{d5}^{\left(\mathrm{aux}\right)} \end{array}\right],e\in R^{10\times 1},$$

**Theorem** **1.** *The task error function e=0 if and only if R=I and t=0, which is proven in Appendix A.*

As mentioned earlier, in the traditional IBVS, it is essential to accurately track each image feature point throughout the entire visual servoing process. This requirement can be stringent and poses challenges, particularly in dynamic environments where feature points may become obscured. In contrast, our PHUVS-based control system can compute the projective homography matrix using a local set of feature points. RANSAC is employed to filter outliers and enhance the robustness of the system to noise. Furthermore, even if the i-th virtual feature point is occluded, we can still determine the scale factor $\gamma_{i}^{\left(\mathrm{aux}\right)}$ and its corresponding error matrix based on Equations (13) and (14). This implies that the loss of certain feature points do not affect the computation of the task function.

If the projective homography matrix is estimated precisely, the vector error $e_{i}$ is equivalent to the position error ${\tilde{\varepsilon }}_{i}^{\left(\mathrm{aux}\right)}$ for the i-th auxiliary feature point. Each element of $e_{i}$ is expressed in the same unit (pixels), ensuring uniformity in magnitude across all components. In the subsequent sections, we use $e_{i}$ as an input signal for the neural network. This consistency simplifies the input quantization process, thus enhancing the effectiveness of the learning process.

We define the system state as:

(17) $$x={\left[\begin{array}{cc} e^{T} & q^{T} \end{array}\right]}^{T}$$

The mapping between the derivative of the new task function and the robot joint velocities is defined as:

(18) $$\dot{e}=J\left(x\right)\dot{q}\;$$

where J is the Jacobian matrix of the entire system.

### 3.2. Collinearity Constraint

**Figure 3:**
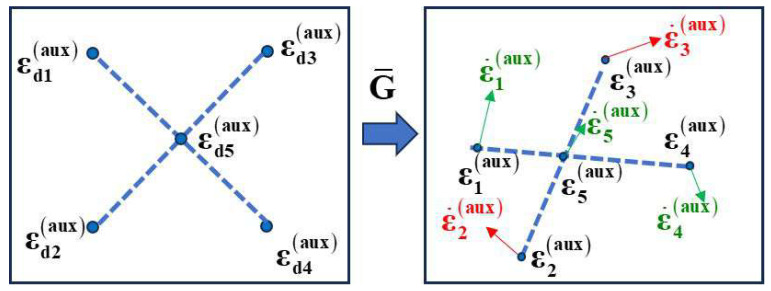
Projective transformation of feature points with collinearity constraints.

In visual servoing systems, utilizing geometric relationships between feature points enhances control performance. As shown in Figure 3, we consider two key collinearity relationships: points $\varepsilon_{d1}^{\left(\mathrm{aux}\right)}$, $\varepsilon_{d4}^{\left(\mathrm{aux}\right)}$ and $\varepsilon_{d5}^{\left(\mathrm{aux}\right)}$ form one collinear set, while $\varepsilon_{d2}^{\left(\mathrm{aux}\right)}$, $\varepsilon_{d3}^{\left(\mathrm{aux}\right)}$ and $\varepsilon_{d5}^{\left(\mathrm{aux}\right)}$ form another. These collinearity properties remain invariant under projective transformations, ensuring that corresponding points in the current configuration ($\varepsilon_{1}^{\left(\mathrm{aux}\right)}$, $\varepsilon_{4}^{\left(\mathrm{aux}\right)}$, $\varepsilon_{5}^{\left(\mathrm{aux}\right)}$) and ($\varepsilon_{2}^{\left(\mathrm{aux}\right)}$, $\varepsilon_{3}^{\left(\mathrm{aux}\right)}$, $\varepsilon_{5}^{\left(\mathrm{aux}\right)}$) maintain the same geometric relationships. The arrows in the figure represent the velocity vectors of the feature points during the servoing process. Based on these collinearity relationships, we derive velocity constraints that preserve the geometric properties throughout the motion.

For collinear points, the following relationships hold:

(19) $$\varepsilon_{5}^{\left(\mathrm{aux}\right)}=\alpha_{1}\varepsilon_{1}^{\left(\mathrm{aux}\right)}+\left(1-\alpha_{1}\right)\varepsilon_{4}^{\left(\mathrm{aux}\right)}$$

(20) $$\varepsilon_{5}^{\left(\mathrm{aux}\right)}=\alpha_{2}\varepsilon_{2}^{\left(\mathrm{aux}\right)}+\left(1-\alpha_{2}\right)\varepsilon_{3}^{\left(\mathrm{aux}\right)}$$

where parameter $\alpha_{1}$ and $\alpha_{2}$ can be calculated as:

(21) $$\alpha_{1}=\frac{{\left(\varepsilon_{5}^{\left(\mathrm{aux}\right)}-\varepsilon_{4}^{\left(\mathrm{aux}\right)}\right)}^{T}\left(\varepsilon_{1}^{\left(\mathrm{aux}\right)}-\varepsilon_{4}^{\left(\mathrm{aux}\right)}\right)}{{\left|\varepsilon_{1}^{\left(\mathrm{aux}\right)}-\varepsilon_{4}^{\left(\mathrm{aux}\right)}\right|}^{2}}$$

(22) $$\alpha_{2}=\frac{{\left(\varepsilon_{5}^{\left(\mathrm{aux}\right)}-\varepsilon_{3}^{\left(\mathrm{aux}\right)}\right)}^{T}\left(\varepsilon_{2}^{\left(\mathrm{aux}\right)}-\varepsilon_{3}^{\left(\mathrm{aux}\right)}\right)}{{\left|\varepsilon_{2}^{\left(\mathrm{aux}\right)}-\varepsilon_{3}^{\left(\mathrm{aux}\right)}\right|}^{2}}$$

Differentiating Equations (19) and (20) with respect to time, we obtain:

(23) $${\dot{\varepsilon }}_{5}^{\left(\mathrm{aux}\right)}=\alpha_{1}{\dot{\varepsilon }}_{1}^{\left(\mathrm{aux}\right)}+\left(1-\alpha_{1}\right){\dot{\varepsilon }}_{4}^{\left(\mathrm{aux}\right)}+{\dot{\alpha }}_{1}\left(\varepsilon_{1}^{\left(\mathrm{aux}\right)}-\varepsilon_{c4}^{\left(\mathrm{aux}\right)}\right)$$

(24) $${\dot{\varepsilon }}_{5}^{\left(\mathrm{aux}\right)}=\alpha_{2}{\dot{\varepsilon }}_{2}^{\left(\mathrm{aux}\right)}+\left(1-\alpha_{2}\right){\dot{\varepsilon }}_{3}^{\left(\mathrm{aux}\right)}+{\dot{\alpha }}_{2}\left(\varepsilon_{2}^{\left(\mathrm{aux}\right)}-\varepsilon_{3}^{\left(\mathrm{aux}\right)}\right)$$

This velocity relationship indicates that the velocity of point 5, ${\dot{\varepsilon }}_{5}^{\left(\mathrm{aux}\right)}$, is a weighted combination of (${\dot{\varepsilon }}_{1}^{\left(\mathrm{aux}\right)}$,${\dot{\varepsilon }}_{4}^{\left(\mathrm{aux}\right)}$) and (${\dot{\varepsilon }}_{2}^{\left(\mathrm{aux}\right)}$, ${\dot{\varepsilon }}_{3}^{\left(\mathrm{aux}\right)}$).

For a static target object, ${\dot{\varepsilon }}_{d1}^{\left(\mathrm{aux}\right)}={\dot{\varepsilon }}_{d2}^{\left(\mathrm{aux}\right)}={\dot{\varepsilon }}_{d3}^{\left(\mathrm{aux}\right)}=\varepsilon_{d4}^{\left(\mathrm{aux}\right)}={\dot{\varepsilon }}_{d5}^{\left(\mathrm{aux}\right)}=0$. In terms of Equations (15), (23) and (24) can be written as:

(25) $$-{\dot{e}}_{5}=-\alpha_{1}{\dot{e}}_{1}-\left(1-\alpha_{1}\right){\dot{e}}_{4}+{\dot{\alpha }}_{1}\left(\varepsilon_{1}^{\left(\mathrm{aux}\right)}-\varepsilon_{4}^{\left(\mathrm{aux}\right)}\right)$$

(26) $$-{\dot{e}}_{5}=-\alpha_{2}{\dot{e}}_{2}-\left(1-\alpha_{2}\right){\dot{e}}_{3}+{\dot{\alpha }}_{2}\left(\varepsilon_{2}^{\left(\mathrm{aux}\right)}-\varepsilon_{3}^{\left(\mathrm{aux}\right)}\right)$$

where ${\dot{e}}_{i}={\dot{\varepsilon }}_{i}^{\left(\mathrm{aux}\right)}-{\dot{\varepsilon }}_{\mathrm{di}}^{\left(\mathrm{aux}\right)}$, i = 1, 2,…5. To eliminate the influence of $\alpha_{1}$ and $\alpha_{2}$ in the last terms, we multiply both sides of the equation by $\left(\varepsilon_{1}^{\left(\mathrm{aux}\right)}-\varepsilon_{4}^{\left(\mathrm{aux}\right)}\right)\in R^{2\times 1}$ and $\left(\varepsilon_{2}^{\left(\mathrm{aux}\right)}-\varepsilon_{3}^{\left(\mathrm{aux}\right)}\right)\in R^{2\times 1}$, yielding:

(27) $$\left(\varepsilon_{1}^{\left(\mathrm{aux}\right)}-\varepsilon_{4}^{\left(\mathrm{aux}\right)}\right)\times \left(-{\dot{e}}_{5}+\alpha_{1}{\dot{e}}_{1}+\left(1-\alpha_{1}\right){\dot{e}}_{4}\right)=0$$

(28) $$\left(\varepsilon_{2}^{\left(\mathrm{aux}\right)}-\varepsilon_{3}^{\left(\mathrm{aux}\right)}\right)\times \left(-{\dot{e}}_{5}+\alpha_{2}{\dot{e}}_{2}+\left(1-\alpha_{2}\right){\dot{e}}_{3}\right)=0$$

These velocity constraints are incorporated into our learning algorithm, ensuring that the neural network updates respect the underlying geometric principles of projective transformations.

### 3.3. Model-Free Jacobian Learning

Traditional methods typically rely on precise mathematical models; however, in practical applications, accurate modeling is often difficult to achieve due to system parameter uncertainties and environmental disturbances. This chapter proposes the use of a CMAC neural network to learn and approximate the system’s Jacobian matrix, enabling more robust and adaptive control strategies. The chapter first introduces the CMAC network structure, then explains the weight learning process. Notably, our weight update approach incorporates geometric constraints of visual features to accelerate the learning process.

#### 3.3.1. CMAC Model

**Figure 4:**
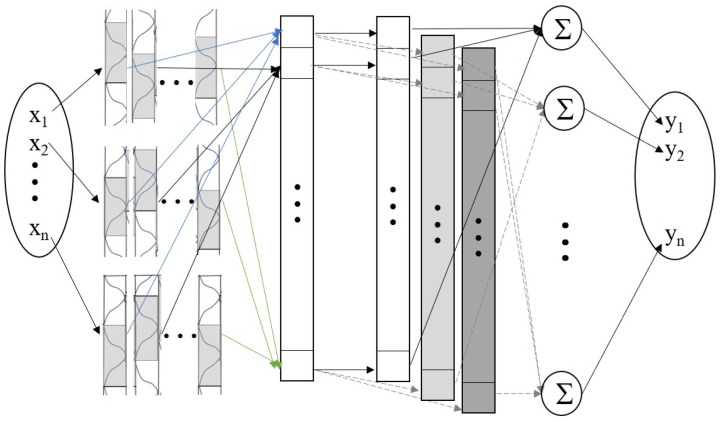
The structure of CMAC network.

The CMAC network is divided into five layers: input layer, association memory space, receptive-field space, weight memory space and output layer, as shown in Figure 4.

**Figure 5:**
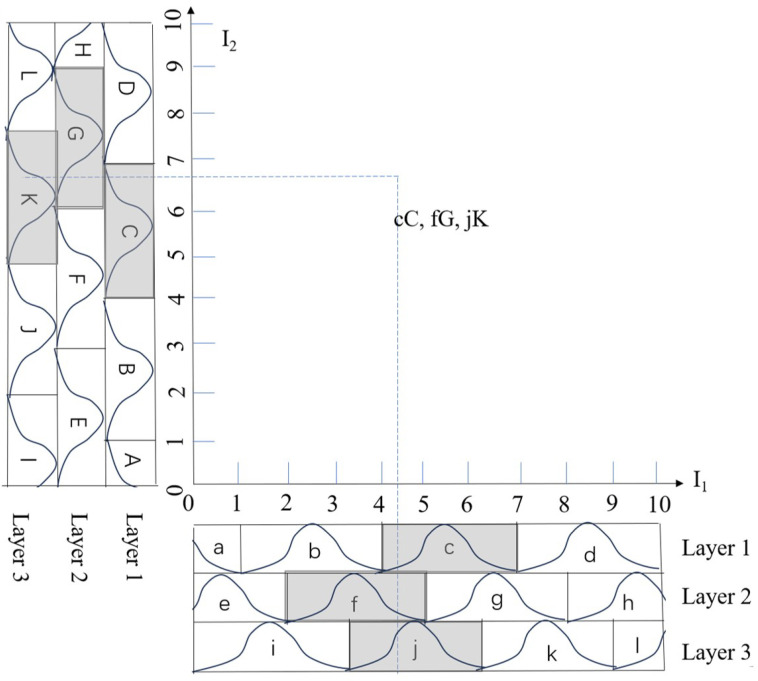
Receptive field organization.

1. Input space:
  In this research, we use the state vector x as the input and employ a neural network to learn the unknown Jacobian matrix J.
  The neural network input dimension is 16×1 ($e\in R^{10\times 1}$ and the robot has six joints: $q\in R^{6\times 1}$). The input vector needs to be quantized into discrete regions according to its corresponding range:
  (29) $$I_{i}=\left\lfloor \frac{x_{i}-x_{i\min}}{x_{i\max}-x_{i\min}}\cdot I_{m}\right\rfloor$$
  where $I_{m}$ represents the maximum index value after quantization, and ⌊·⌋ denotes the floor function, ensuring that $I_{i}$ is an integer. And $x_{i\min}$ and $x_{i\max}$ represent the minimum and maximum values of ith element of state vector x. Figure 5 illustrates a CMAC neural network with two-dimensional input, where each input is discretized into 10 regions: $I_{m}=10$.
2. Association memory space:
  Each input dimension is uniformly partitioned into several discrete regions. Each complete block includes three adjacent regions. In Figure 5, input $x_{1}$ and $x_{2}$ have their regions grouped into blocks (a, b, c, d) and (A, B, C, D), respectively.
  The network creates multiple layers by shifting the block boundaries. For example, layer 2 contains blocks D, E, F for $x_{1}$ and blocks d, e, f for $x_{2}$, formed by shifting the original blocks by one region. Subsequent layer 3 follows the same pattern with additional shifts. Each block is represented by a Gaussian membership function:
  (30) $$\psi_{ik}=\exp\left(\frac{-{\left(I_{i}-m_{ik}\right)}^{2}}{\sigma_{ik}^{2}}\right),k\in \left[1,n_{b}\right]$$
  where *k* denotes the *k*-th block and $n_{b}$ represents the total number of blocks for input dimension $I_{i}$. The parameters $m_{ik}$ and $\sigma_{ik}$ represent the center and width of the Gaussian function for the *k*-th block, respectively.
3. Receptive-field space:
  The receptive field refers to the region in the input space that can activate specific neurons or memory cells in the network. A key feature of the CMAC network is the use of overlapping receptive fields. This means that a single input can simultaneously activate multiple memory cells, creating a distributed representation. The degree of overlap affects the network’s smoothness and generalization capability; highly overlapping receptive fields produce smoother function approximations, while less overlap produces more localized responses. As shown in Figure 5, point p in space falls within blocks aB, fD, and Ee, activating the corresponding memory cells. The multidimensional receptive-field function is defined as
  (31) $$b_{k}=\underset{i=1}{\overset{n}{\Pi }}\;\psi_{ik}=\exp\left(\sum_{i=1}^{n}\frac{-{\left(I_{i}-m_{ik}\right)}^{2}}{\sigma_{ik}^{2}}\right),k=1,2\cdots n_{l}$$
  where $n_{l}$ represents the number of layers in the receptive field space.
4. Weight memory space and output layer:
  Each receptive field space is connected to the weighted memory W. The i-th output of the neural network is:
  (32) $$y_{i}=\sum_{k=1}^{n_{l}}w_{ik}b_{k}=w_{i}^{T}b$$
  where $w_{i}={\left[\begin{array}{cccc} w_{i1} & w_{i2} & \cdots & w_{in_{l}} \end{array}\right]}^{T}$ and $b={\left[\begin{array}{cccc} b_{1} & b_{2} & \cdots & b_{n_{l}} \end{array}\right]}^{T}$.

#### 3.3.2. Weight Update of Neural Network

The discrete form of the Equation (18) can be written as:

(33) $$\Delta e\left(t_{i}\right)=J\left(t_{i}\right)\Delta q\left(t_{i}\right)$$

where the subscript i represents the i-th time step. $\Delta q\in R^{n_{q}\times 1}$ represents the joint increment over the sampling interval. Next, the state vector x is used as input to approximate the Jacobian matrix using the CMAC network.

(34) $$J=\left[\begin{array}{cccc} w_{1,1}^{T}b\left(x\left(t_{i}\right)\right) & w_{1,2}^{T}b\left(x\left(t_{i}\right)\right) & \cdots & w_{1,n_{q}}^{T}b\left(x\left(t_{i}\right)\right)\\ w_{2,1}^{T}b\left(x\left(t_{i}\right)\right) & w_{2,2}^{T}b\left(x\left(t_{i}\right)\right) & \cdots & w_{2,n_{q}}^{T}b\left(x\left(t_{i}\right)\right)\\ \vdots & \vdots & \vdots & \vdots \\ w_{10,1}^{T}b\left(x\left(t_{i}\right)\right) & w_{10,2}^{T}b\left(x\left(t_{i}\right)\right) & \cdots & w_{10,n_{q}}^{T}b\left(x\left(t_{i}\right)\right) \end{array}\right]\;+\sigma_{J}$$

where w are constant matrices of ideal network weights and $w_{k,m}\in R^{n_{l}\times 1}$ is used to approximate the i-th row and jth column element of the Jacobian matrix $j_{km}$, and b is the vector of receptive-field functions. The approximation error of the neural network is denoted as $\sigma_{J}$.

It is important to note that the estimated incremental task error

$$\Delta e={\left[\begin{array}{cccc} \Delta e_{1}^{T} & \Delta e_{2}^{T} & \cdots & \Delta e_{5}^{T} \end{array}\right]}^{T}$$

consists of five 2D vectors, where each $\Delta e_{i}\in R^{2\times 1}$. To distinguish between these components, we use superscript notation to denote the k-th element of Δe: $\Delta e^{k}$. Thus, we have:

(35) $$\Delta e^{k}\left(t_{i}\right)=\sum_{m=1}^{n_{q}}j_{km}\left(t_{i}\right)\Delta q_{m}\left(t_{i}\right)+\sigma_{k}=\Phi^{T}w_{k}+\sigma_{k}$$

where $w_{k}={\left[\begin{array}{cccc} w_{k,1}^{T} & w_{k,2}^{T} & \cdots & w_{k,n_{q}}^{T} \end{array}\right]}^{T}$ and $\sigma_{k}$ is the approximation error of the neural network. $\Phi^{T}=\left[\begin{array}{cccc} b^{T}\left(x\left(t_{i}\right)\right)\Delta q_{1} & b^{T}\left(x\left(t_{i}\right)\right)\Delta q_{2} & \cdots & b^{T}\left(x\left(t_{i}\right)\right)\Delta q_{n_{q}} \end{array}\right]$, and $\Delta q_{m}$ represents the *m*-th element of Δq.

Δe is further expressed as shown below:

(36) $$\Delta e\left(t_{i}\right)=\sum_{m=1}^{n_{q}}j_{m}\left(t_{i}\right)\Delta q_{m}\left(t_{i}\right)=\Omega^{T}w+\sigma$$

where $j_{m}$ represents the m-th column of J. $\sigma ={\left[\begin{array}{cccc} \sigma_{1} & \sigma_{2} & \cdots & \sigma_{10} \end{array}\right]}^{T}$ is the approximation error term introduced by the neural network model. $\Omega^{T}=\left[\begin{array}{cccc} \Phi^{T} & 0 & \cdots & 0\\ 0 & \Phi^{T} & \cdots & 0\\ 0 & 0 & \ddots & 0\\ 0 & 0 & \cdots & \Phi^{T} \end{array}\right]$ and $w={\left[\begin{array}{cccc} w_{1}^{T} & w_{2}^{T} & \cdots & w_{10}^{T} \end{array}\right]}^{T}$.

**Assumption** **1.** *The approximation error **σ** is assumed to be upper bounded:*

$$∥\sigma \left(t\right)∥\leq \sigma_{M}$$

*where $\sigma_{M}$ is a finite positive constant. In addition, under a properly designed neural network architecture, **σ** can be made sufficiently small.*

However, w is unknown. Using the estimated weights, the *m*-th column and the *k*-th row of the estimated Jacobian matrix can be given by: ${\hat{j}}_{km}\left(t_{i}\right)={\hat{w}}_{k,m}\left(t_{i}\right)b\left(x\left(t_{i}\right)\right)$ where ${\hat{w}}_{k,m}$ is the estimated value of desired $w_{k,m}$.

The estimated value of $\Delta e^{k}$ is:

(37) $$\Delta {\hat{e}}^{k}\left(t_{i}\right)=\Phi^{T}{\hat{w}}_{k}$$

The estimated incremental change in task error is:

(38) $$\Delta \hat{e}\left(t_{i}\right)=\hat{J}\left(t_{i}\right)\Delta q\left(t_{i}\right)=\Omega^{T}\hat{w}$$

To incorporate geometric constraints into the learning process, we leverage the velocity relationships between collinear feature points defined in Equations (27) and (28). The Jacobian matrix estimated by our neural network must satisfy the following constraints:

(39) $$h_{1}=f_{1}\left(\Delta \hat{e}\right)=\left(\varepsilon_{1}^{\left(\mathrm{aux}\right)}-\varepsilon_{4}^{\left(\mathrm{aux}\right)}\right)\times \left(-\Delta {\hat{e}}_{5}+\alpha_{1}\Delta {\hat{e}}_{1}+\left(1-\alpha_{1}\right)\Delta {\hat{e}}_{4}\right)$$

(40) $$h_{2}=f_{2}\left(\Delta \hat{e}\right)=\left(\varepsilon_{2}^{\left(\mathrm{aux}\right)}-\varepsilon_{3}^{\left(\mathrm{aux}\right)}\right)\times \left(-\Delta {\hat{e}}_{5}+\alpha_{2}\Delta {\hat{e}}_{2}+\left(1-\alpha_{2}\right)\Delta {\hat{e}}_{3}\right)$$

We then define a loss function that incorporates both prediction accuracy and geometric constraints:

(41) $$\min L=\min\left(\frac{1}{2}{\left(\Delta e-\Delta \hat{e}\right)}^{T}\left(\Delta e-\Delta \hat{e}\right)+\frac{\lambda }{2}\sum_{j=1}^{2}h_{j}^{2}\right)$$

Then, the weights of neural network are updated by:

(42) $$\hat{w}\left(t_{i+1}\right)=\hat{w}\left(t_{i}\right)+\beta \frac{\partial L}{\partial \hat{w}}=\hat{w}\left(t_{i}\right)-\beta \left(\Omega \left(t_{i}\right)\Delta \tilde{e}\left(t_{i}\right)-\sum_{j=1}^{2}\lambda h_{j}\frac{\partial h_{j}}{\Delta \hat{w}}\right)$$

where β represents the learning rate parameter for the neural network weight update process, and $\Delta \tilde{e}=\Delta e-\Delta \hat{e}$ is the estimation error.

### 3.4. Controller Design

The control law of a robot is defined as:

(43) $$u=-\eta {\hat{J}}^{+}\left(\left.x\right|\hat{w}\right)e$$

where η is a positive value.

**Figure 6:**
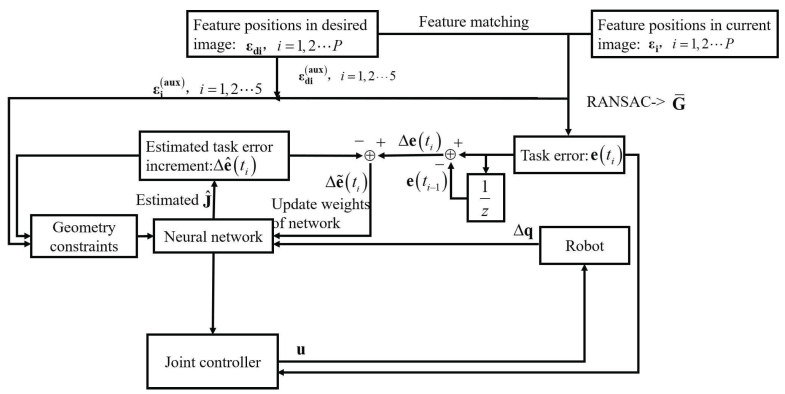
Control flow diagram.

Figure 6 illustrates the control flow diagram. The process begins with image acquisition and feature point detection. Matched feature pairs $\left\{\varepsilon_{i},\varepsilon_{di}\right\},\;i=1,2,\cdots ,P$ are utilized to estimate the projective homography matrix $\bar{G}$ using the RANSAC algorithm. Based on $\bar{G}$, the task error vector e and a set of virtual feature points $\varepsilon_{i}^{\left(\mathrm{aux}\right)}$ are computed according to Equations (12)–(16).

Next, the estimated system state vector, which includes the task error e and the joint position q, is input to the CMAC neural network. The network predicts the task error increment $\Delta \hat{e}$ and estimates the Jacobian matrix. Importantly, the geometry constraint module is activated after this estimation phase. It incorporates $\Delta \hat{e}$ and the virtual feature points to compute constraint terms (see Equations (39) and (40)), which are then embedded into the neural network’s weight update rule (Equation (42)) to preserve geometric consistency, such as point collinearity under projective transformations.

Finally, the joint controller utilizes the estimated Jacobian and task error to compute the control command u, as described in Equation (43), which is then sent to the robot actuators. The entire procedure operates in a closed-loop fashion, with the visual feedback continually guiding subsequent control iterations.

## 4. Stability Analysis

In this section, we establish the stability of the proposed geometry-constrained learning-based visual servoing controller.

### 4.1. Geometric Constraint Relationships

Let us define the weight estimation error as $\tilde{w}=w-\hat{w}$, where w represents the ideal weights. According to Equations (35) and (37), we have:

(44) $$\Delta {\tilde{e}}^{k}=\Delta e^{k}-\Delta {\hat{e}}^{k}=\Phi^{T}{\tilde{w}}_{k}+\sigma_{k}$$

Similarly, from Equations (36) and (38), we can derive:

(45) $$\Delta \tilde{e}=\Delta e-\Delta \hat{e}=\Omega^{T}\tilde{w}+\sigma$$

For the actual error increment Δe, the geometric constraints must be satisfied, i.e., $h_{i}\left(\Delta e\right)=0$. We establish the relationship between these constraint equations and the estimation error of the task error increment:

(46) $$\begin{array}{cc} h_{1}\left(\Delta \hat{e}\right)-h_{1}\left(\Delta e\right) & =h_{1}\\ \; & =\left(\varepsilon_{1}^{\left(\mathrm{aux}\right)}-\varepsilon_{4}^{\left(\mathrm{aux}\right)}\right)\times \left(-\Delta e_{5}+\alpha_{1}\Delta e_{1}+\left(1-\alpha_{1}\right)\Delta e_{4}\right)\\ \; & -\left(\varepsilon_{1}^{\left(\mathrm{aux}\right)}-\varepsilon_{4}^{\left(\mathrm{aux}\right)}\right)\times \left(-\Delta {\hat{e}}_{5}+\alpha_{1}\Delta {\hat{e}}_{1}+\left(1-\alpha_{1}\right)\Delta {\hat{e}}_{4}\right)\\ \; & =\left(\varepsilon_{1}^{\left(\mathrm{aux}\right)}-\varepsilon_{4}^{\left(\mathrm{aux}\right)}\right)\times \left(-\Delta {\tilde{e}}_{5}+\alpha_{1}\Delta {\tilde{e}}_{1}+\left(1-\alpha_{1}\right)\Delta {\tilde{e}}_{4}\right) \end{array}$$

Similarly, for the second constraint:

(47) $$\begin{array}{cc} h_{2} & =\left(\varepsilon_{2}^{\left(\mathrm{aux}\right)}-\varepsilon_{3}^{\left(\mathrm{aux}\right)}\right)\times \left(-\Delta {\tilde{e}}_{5}+\alpha_{2}\Delta {\tilde{e}}_{2}+\left(1-\alpha_{2}\right)\Delta {\tilde{e}}_{3}\right) \end{array}$$

It should be noted that $\Delta {\tilde{e}}_{i}={\left[\Delta {\tilde{e}}^{2i-1}\;\Delta {\tilde{e}}^{2i}\right]}^{T}$ is a two-dimensional vector containing the (2i−1)-th and 2i-th elements of $\Delta \tilde{e}$. The constraints $h_{1}$ and $h_{2}$ can be reformulated as:

(48) $$\begin{array}{cc} h_{1} & =\mu_{u}\left(-\Delta {\tilde{e}}^{9}+\alpha_{1}\Delta {\tilde{e}}^{1}+\left(1-\alpha_{1}\right)\Delta {\tilde{e}}^{7}\right)\\ \; & -\mu_{v}\left(-\Delta {\tilde{e}}^{10}+\alpha_{1}\Delta {\tilde{e}}^{2}+\left(1-\alpha_{1}\right)\Delta {\tilde{e}}^{8}\right)\\ \; & =\mu_{u}\left(-\Phi^{T}{\tilde{w}}_{9}-\sigma_{9}+\alpha_{1}\Phi^{T}{\tilde{w}}_{1}+\alpha_{1}\sigma_{1}+\left(1-\alpha_{1}\right)\Phi^{T}{\tilde{w}}_{7}+\left(1-\alpha_{1}\right)\sigma_{7}\right)\\ \; & -\mu_{v}\left(-\Phi^{T}{\tilde{w}}_{10}-\sigma_{10}+\alpha_{1}\Phi^{T}{\tilde{w}}_{2}+\alpha_{1}\sigma_{2}+\left(1-\alpha_{1}\right)\Phi^{T}{\tilde{w}}_{8}+\left(1-\alpha_{1}\right)\sigma_{8}\right)\\ \; & =n_{1}^{T}\tilde{w}+\mu_{u}\left(-\sigma_{9}+\alpha_{1}\sigma_{1}+\left(1-\alpha_{1}\right)\sigma_{7}\right)-\mu_{v}\left(-\sigma_{10}+\alpha_{1}\sigma_{2}+\left(1-\alpha_{1}\right)\sigma_{8}\right)\\ \; & =n_{1}^{T}\tilde{w}+\sigma_{h1} \end{array}$$

(49) $$\begin{array}{cc} h_{2} & =\kappa_{u}\left(-\Delta {\tilde{e}}^{9}+\alpha_{2}\Delta {\tilde{e}}^{3}+\left(1-\alpha_{2}\right)\Delta {\tilde{e}}^{5}\right)\\ \; & +\kappa_{v}\left(-\Delta {\tilde{e}}^{10}+\alpha_{2}\Delta {\tilde{e}}^{4}+\left(1-\alpha_{2}\right)\Delta {\tilde{e}}^{6}\right)\\ \; & =\kappa_{u}\left(-\Phi^{T}{\tilde{w}}_{9}-\sigma_{9}+\alpha_{2}\Phi^{T}{\tilde{w}}_{3}+\alpha_{2}\sigma_{3}+\left(1-\alpha_{2}\right)\Phi^{T}{\tilde{w}}_{5}+\left(1-\alpha_{2}\right)\sigma_{5}\right)\\ \; & -\kappa_{v}\left(-\Phi^{T}{\tilde{w}}_{10}-\sigma_{10}+\alpha_{2}\Phi^{T}{\tilde{w}}_{4}+\alpha_{2}\sigma_{4}+\left(1-\alpha_{2}\right)\Phi^{T}{\tilde{w}}_{6}+\left(1-\alpha_{2}\right)\sigma_{6}\right)\\ \; & =n_{2}^{T}\tilde{w}+\kappa_{u}\left(-\sigma_{9}+\alpha_{2}\sigma_{3}+\left(1-\alpha_{2}\right)\sigma_{5}\right)-\kappa_{v}\left(-\sigma_{10}+\alpha_{2}\sigma_{4}+\left(1-\alpha_{2}\right)\sigma_{6}\right)\\ \; & =n_{2}^{T}\tilde{w}+\sigma_{h2} \end{array}$$

where $\sigma_{h1}$ and $\sigma_{h2}$ represent the combined approximation errors in the constraint equations. $\mu_{u}$ and $\mu_{v}$ are the first and second elements of $\left(\varepsilon_{1}^{\left(\mathrm{aux}\right)}-\varepsilon_{4}^{\left(\mathrm{aux}\right)}\right)$, respectively. Similarly, $\kappa_{u}$ and $\kappa_{v}$ are the first and second elements of $\left(\varepsilon_{2}^{\left(\mathrm{aux}\right)}-\varepsilon_{3}^{\left(\mathrm{aux}\right)}\right)$.

The vectors $n_{1}$ and $n_{2}$ are defined as:

(50) $$\begin{array}{cc} n_{1}^{T} & =\\ \; & \left[\begin{array}{cccccccccc} \mu_{u}\alpha_{1}\Phi^{T} & -\mu_{v}\alpha_{1}\Phi^{T} & 0 & 0 & 0 & 0 & \mu_{u}\left(1-\alpha_{1}\right)\Phi^{T} & -\mu_{v}\left(1-\alpha_{1}\right)\Phi^{T} & -\mu_{u}\Phi^{T} & \mu_{v}\Phi^{T} \end{array}\right]\\ n_{2}^{T} & =\\ \; & \left[\begin{array}{cccccccccc} 0 & 0 & \mu_{u}\alpha_{2}\Phi^{T} & -\mu_{v}\alpha_{2}\Phi^{T} & \mu_{u}\left(1-\alpha_{2}\right)\Phi^{T} & -\mu_{v}\left(1-\alpha_{2}\right)\Phi^{T} & 0 & 0 & -\mu_{u}\Phi^{T} & \mu_{v}\Phi^{T} \end{array}\right] \end{array}$$

The geometric constraints contribute to the neural network weight update rule as shown in Equation (42). The second term in this equation can be reformulated as:

(51) $$\begin{array}{cc} \sum_{j=1}^{2}h_{j}\frac{\partial h_{j}}{\partial \hat{w}} & =h_{1}\frac{\partial h_{1}}{\partial \tilde{w}}\frac{\partial \tilde{w}}{\partial \hat{w}}+h_{2}\frac{\partial h_{2}}{\partial \tilde{w}}\frac{\partial \tilde{w}}{\partial \hat{w}}\\ \; & =\left(n_{1}^{T}\tilde{w}+\sigma_{h1}\right)\left(-n_{1}\right)+\left(n_{2}^{T}\tilde{w}+\sigma_{h2}\right)\left(-n_{2}\right)\\ \; & =-n_{1}n_{1}^{T}\tilde{w}-\sigma_{h1}n_{1}-n_{2}n_{2}^{T}\tilde{w}-\sigma_{h2}n_{2}\\ \; & =-N\tilde{w}-\sigma_{N} \end{array}$$

where $N=n_{1}n_{1}^{T}+n_{2}n_{2}^{T}$ and $\sigma_{N}=\sigma_{h1}n_{1}+\sigma_{h2}n_{2}$ represent the combined approximation error effect.

The weight error dynamics can thus be expressed as:

(52) $$\begin{array}{cc} \tilde{w}\left(t_{i+1}\right) & =\tilde{w}\left(t_{i}\right)-\beta \left(\Omega \left(t_{i}\right)\Delta \tilde{e}\left(t_{i}\right)-\lambda \sum_{j=1}^{2}h_{j}\frac{\partial h_{j}}{\partial \hat{w}}\right)\\ \; & =\tilde{w}\left(t_{i}\right)-\beta \left(\Omega \left(t_{i}\right)\left(\Omega^{T}\tilde{w}+\sigma \right)+\lambda N\tilde{w}+\lambda \sigma_{N}\right)\\ \; & =\tilde{w}\left(t_{i}\right)-\beta \Omega \left(t_{i}\right)\Omega^{T}\tilde{w}-\beta \Omega \left(t_{i}\right)\sigma -\beta \lambda N\tilde{w}-\beta \lambda \sigma_{N}\\ \; & =\left(I-\beta M-\beta \lambda N\right)\tilde{w}\left(t_{i}\right)-\beta \Omega \left(t_{i}\right)\sigma -\beta \lambda \sigma_{N} \end{array}$$

where $M=\Omega \left(t_{i}\right)\Omega^{T}$.

### 4.2. Lyapunov Stability Analysis

To analyze the stability of the learning process, we define a positive definite Lyapunov function candidate:

(53) $$V\left(t_{i}\right)=\frac{1}{2}{\tilde{w}}^{T}\left(t_{i}\right)\tilde{w}\left(t_{i}\right)$$

The change in this Lyapunov function between consecutive time steps is:

(54) $$\begin{array}{cc} \Delta V & =V\left(t_{i+1}\right)-V\left(t_{i}\right)\\ \; & =\frac{1}{2}{\tilde{w}}^{T}\left(t_{i+1}\right)\tilde{w}\left(t_{i+1}\right)-\frac{1}{2}{\tilde{w}}^{T}\left(t_{i}\right)\tilde{w}\left(t_{i}\right) \end{array}$$

Substituting the weight error dynamics:

(55) $$\begin{array}{cc} \Delta V & =\frac{1}{2}{\left[\left(I-\beta M-\beta \lambda N\right)\tilde{w}\left(t_{i}\right)-\beta \Omega \left(t_{i}\right)\sigma -\beta \lambda \sigma_{N}\right]}^{T}\\ \; & \;\cdot [\left(I-\beta M-\beta \lambda N\right)\tilde{w}\left(t_{i}\right)-\beta \Omega \left(t_{i}\right)\sigma -\beta \lambda \sigma_{N}]\\ \; & \;-\frac{1}{2}{\tilde{w}}^{T}\left(t_{i}\right)\tilde{w}\left(t_{i}\right) \end{array}$$

where $M=\Omega \left(t_{i}\right)\Omega^{T}\left(t_{i}\right)$ and $N=n_{1}n_{1}^{T}+n_{2}n_{2}^{T}$ are both positive semi-definite matrices.

Equation (55) can be simplified as:

(56) $$\begin{array}{cc} \Delta V & =-\beta {\tilde{w}}^{T}\left(t_{i}\right)\left(M+\lambda N\right)\tilde{w}-\beta {\tilde{w}}^{T}\left(t_{i}\right)\left(\Omega \left(t_{i}\right)\sigma +\lambda \sigma_{N}\right)\\ \; & \;+\frac{1}{2}\beta^{2}{\tilde{w}}^{T}\left(t_{i}\right){\left(M+\lambda N\right)}^{2}\tilde{w}\\ \; & \;+\beta^{2}{\tilde{w}}^{T}\left(t_{i}\right)\left(M+\lambda N\right)\left(\Omega \left(t_{i}\right)\sigma +\lambda \sigma_{N}\right)\\ \; & \;+\frac{1}{2}\beta^{2}{∥\Omega \left(t_{i}\right)\sigma +\lambda \sigma_{N}∥}^{2}\\ \; & =-\beta {\tilde{w}}^{T}\left(t_{i}\right)\left[\left(M+\lambda N\right)-\frac{\beta }{2}{\left(M+\lambda N\right)}^{2}\right]\tilde{w}\\ \; & \;-\beta {\tilde{w}}^{T}\left(t_{i}\right)\left(I-\beta \left(M+\lambda N\right)\right)\left(\Omega \left(t_{i}\right)\sigma +\lambda \sigma_{N}\right)\\ \; & \;+\frac{1}{2}\beta^{2}{∥\Omega \left(t_{i}\right)\sigma +\lambda \sigma_{N}∥}^{2} \end{array}$$

Let us define $P=\left(M+\lambda N\right)-\frac{\beta }{2}{\left(M+\lambda N\right)}^{2}$ and Q=I−β(M+λN). Then:

(57) $$\begin{array}{cc} \Delta V & =-\beta {\tilde{w}}^{T}\left(t_{i}\right)P\tilde{w}-\beta {\tilde{w}}^{T}\left(t_{i}\right)Q\left(\Omega \left(t_{i}\right)\sigma +\lambda \sigma_{N}\right)\\ \; & \;+\frac{1}{2}\beta^{2}{∥\Omega \left(t_{i}\right)\sigma +\lambda \sigma_{N}∥}^{2} \end{array}$$

Based on Assumption 1, and noting that both the joint increment Δq and the activation of the receptive field b(x) are bounded, it follows that $\Omega (t_{i})$ is also bounded. Consequently, there exists a small positive constant ϵ>0 such that:

(58) $$∥\Omega \left(t_{i}\right)\sigma +\lambda \sigma_{N}∥\leq \epsilon ,\;\forall t_{i}$$

To rigorously ensure stability, we assume the following Persistent Excitation (PE) condition:

**Assumption** **2** **(Persistent Excitation).** *There exists a positive constant $\gamma_{\min}>0$ and a finite time interval T>0, such that for all t:*

(59) $$\frac{1}{T}\int_{t}^{t+T}\Omega \left(\tau \right)\Omega^{T}\left(\tau \right)d\tau \geq \gamma_{\min}I$$

*Under this assumption, define the average matrix as:*

(60) $$M_{avg}=\frac{1}{T}\int_{t}^{t+T}M\left(\tau \right)d\tau \geq \gamma_{\min}I$$

*Hence, the averaged matrix $M_{avg}+\lambda N$ is strictly positive definite, ensuring:*

(61) $$x^{T}\left(M_{avg}+\lambda N\right)x\geq \gamma_{\min}{∥x∥}^{2},\;\forall x\neq 0$$

*Selecting the learning rate β to be sufficiently small:*

(62) $$0<\beta <\frac{2}{\lambda_{\max}\left(M_{avg}+\lambda N\right)}$$

*the Lyapunov function increment becomes:*

(63) $$\Delta V\leq -\beta \gamma_{\min}∥\tilde{w}\left(t_{i}\right){∥}^{2}+\beta ∥\tilde{w}\left(t_{i}\right)∥\epsilon +\frac{1}{2}\beta^{2}\epsilon^{2}$$

Therefore, the weight estimation error $\tilde{w}\left(t_{i}\right)$ is uniformly ultimately bounded, and the system error converges to a small neighborhood of the origin, ensuring robust convergence and stability under the persistent excitation condition.

### 4.3. Convergence to Desired Pose

In practice, the estimated Jacobian $\hat{J}$ differs from the true Jacobian J due to learning or modeling errors. We define the Jacobian estimation error as:

(64) $$\tilde{J}\left(t_{k}\right)=J\left(t_{k}\right)-\hat{J}\left(t_{k}\right)$$

Assume the control update is given by $u\left(t_{k}\right)=-\eta {\hat{J}}^{+}\left(t_{k}\right)e\left(t_{k}\right)$. Then, the error propagation becomes:

(65) $$\begin{array}{cc} e(t_{k+1}) & =e\left(t_{k}\right)+J\left(t_{k}\right)u\left(t_{k}\right)\\ \; & =e\left(t_{k}\right)-\eta J\left(t_{k}\right){\hat{J}}^{+}\left(t_{k}\right)e\left(t_{k}\right)\\ \; & =\left(I-\eta S(t_{k})\right)e\left(t_{k}\right) \end{array}$$

where we define the matrix $S=J{\hat{J}}^{+}=\left(\hat{J}+\tilde{J}\right){\hat{J}}^{+}=I+\delta_{s}$.

Given that the neural network estimation error has been proven to converge, and with an appropriate neural network structure design along with sufficient pre-training data to obtain quality initial weights, it is reasonable to assume that $\delta_{s}$ is bounded. There exists a constant $\sigma_{\Delta s}$ such that:

$$∥\delta_{s}{∥}_{2}\leq \sigma_{\Delta s}$$

Define the Lyapunov function:

(66) $$V\left(t_{k}\right)=\frac{1}{2}{∥e\left(t_{k}\right)∥}^{2}$$

The difference is:

(67) $$\begin{array}{cc} V\left(t_{k+1}\right)-V\left(t_{k}\right)\; & =\frac{1}{2}∥e\left(t_{k+1}\right){∥}^{2}-\frac{1}{2}{∥e\left(t_{k}\right)∥}^{2}\\ \; & =\frac{1}{2}{∥\left(I-\eta S(t_{k})\right)e\left(t_{k}\right)∥}^{2}-\frac{1}{2}{∥e\left(t_{k}\right)∥}^{2}\\ \; & =-\eta e^{T}\left(t_{k}\right)S\left(t_{k}\right)e\left(t_{k}\right)+\frac{\eta^{2}}{2}{∥S\left(t_{k}\right)e\left(t_{k}\right)∥}^{2} \end{array}$$

To ensure convergence (i.e., $V\left(t_{k+1}\right)-V\left(t_{k}\right)<0$), we require that $\eta e^{T}Se>\frac{\eta^{2}}{2}{∥Se∥}^{2}$. This inequality holds for all e≠0 if the matrix $D=S+\frac{\eta }{2}S^{T}S$ is positive definite. We now derive a conservative bound on η to guarantee the positive definiteness of D.

For any unit vector $x\in R^{n}$, we have:

(68) $$x^{T}Sx=x^{T}\left(I+\delta_{s}\right)x=1+x^{T}\delta_{s}x\geq 1-\sigma_{\Delta s}$$

(69) $$\lambda_{\min}\left(S^{T}S\right)=\lambda_{\min}^{2}\left(S\right)\geq {\left(1-\sigma_{\Delta s}\right)}^{2}$$

Hence:

(70) $$x^{T}Dx=x^{T}Sx+\frac{\eta }{2}x^{T}S^{T}Sx\geq \left(1-\sigma_{\Delta s}\right)+\frac{\eta }{2}{\left(1-\sigma_{\Delta s}\right)}^{2}$$

To ensure $x^{T}Dx>0$, we require:

(71) $$\left(1-\sigma_{\Delta s}\right)+\frac{\eta }{2}{\left(1-\sigma_{\Delta s}\right)}^{2}>0\;\Rightarrow \;\eta >\frac{-2(1-\sigma_{\Delta s})}{{\left(1-\sigma_{\Delta s}\right)}^{2}}$$

When the Jacobian estimation error is relatively small ($\sigma_{\Delta s}<1$), the system can maintain stability across a wide range of control gains. When the error becomes too large ($\sigma_{\Delta s}>1$), a larger control gain is required to maintain stability, but this may lead to other issues (such as excessive oscillation).

Combined with Theorem 1, which shows that e=0 if and only if R=I and t=0, it follows that the camera pose converges to the desired pose. The proposed controller thus ensures robust convergence despite approximation errors in the neural network.

## 5. Simulation and Experiment Results

### 5.1. Pre-Training Process

To ensure efficient initialization and promote global convergence during the visual servoing task, a pre-training phase is conducted for the neural network. In this phase, the robot performs small randomized motions around its initial configuration. During these exploratory movements, the system collects training data consisting of system states x, the corresponding joint displacements, and the incremental task errors.

The collected system state data are denoted as $X=\left\{x\left(t_{0}\right),x\left(t_{1}\right),\cdots ,x\left(t_{n_{s}}\right)\right\}$, where each sample may activate a distinct subset of local receptive fields within the neural network. The union of all receptive fields activated by the sample set X is represented by $b_{s}\left(X\right)\in R^{n_{l}\times 1}$, where $n_{l}$ denotes the total number of receptive fields. Each entry in $b_{s}\left(X\right)$ indicates whether the corresponding receptive field is activated by any sample in the dataset.

The activation matrix A(X) is defined as:

$$A\left(X\right)=\left[\begin{array}{ccc} b_{s}^{⊤}\left(x\left(t_{0}\right)\right)\Delta q_{1}\left(t_{0}\right) & \cdots & b_{s}^{⊤}\left(x\left(t_{0}\right)\right)\Delta q_{n_{q}}\left(t_{0}\right)\\ b_{s}^{⊤}\left(x\left(t_{1}\right)\right)\Delta q_{1}\left(t_{1}\right) & \cdots & b_{s}^{⊤}\left(x\left(t_{1}\right)\right)\Delta q_{n_{q}}\left(t_{1}\right)\\ \vdots & \ddots & \vdots \\ b_{s}^{⊤}\left(x\left(t_{n_{s}}\right)\right)\Delta q_{1}\left(t_{n_{s}}\right) & \cdots & b_{s}^{⊤}\left(x\left(t_{n_{s}}\right)\right)\Delta q_{n_{q}}\left(t_{n_{s}}\right) \end{array}\right],\;A\in R^{n_{s}\times \left(n_{q}n_{l}\right)}.$$

where each row of A corresponds to a training sample, and each column corresponds to the influence of a specific receptive field on a particular joint.

The k-th element of incremental task error Δe is expressed as:

(72) $$\Delta e^{k,\mathrm{sample}}=A\left(X\right)w_{k},$$

where $w_{k}\in R^{n_{q}n_{l}\times 1}$ denotes the weight vector of the neural network associated with the *k*-th row of the estimated Jacobian matrix. The vector $\Delta e^{k,\mathrm{sample}}\in R^{n_{s}\times 1}$ contains the incremental task errors for all samples in the dataset, and is defined as:

$$\Delta e^{k,\mathrm{sample}}={\left[\Delta e^{k}\left(t_{0}\right),\;\Delta e^{k}\left(t_{1}\right),\;\cdots ,\;\Delta e^{k}\left(t_{n_{s}}\right)\right]}^{⊤}.$$

The weights of neural network are initially estimated by solving the linear regression problem:

(73) $$w_{k}={\left(A^{T}A\right)}^{-1}A^{T}\Delta e_{k,\mathrm{sample}},\;k=1,2,\cdots ,10.$$

This pre-training strategy effectively initializes the network weights, thus increasing the chances of achieving global convergence.

### 5.2. Simulations

#### 5.2.1. Performance Analysis

To validate our algorithm, we conducted simulations using a 6-DOF Universal Robot with a camera mounted on its end effector. Recognizing that unsuitable initial weights in the neural network could cause the control error to settle on a local minimum, we implemented pre-training on the neural network.

**Figure 7:**
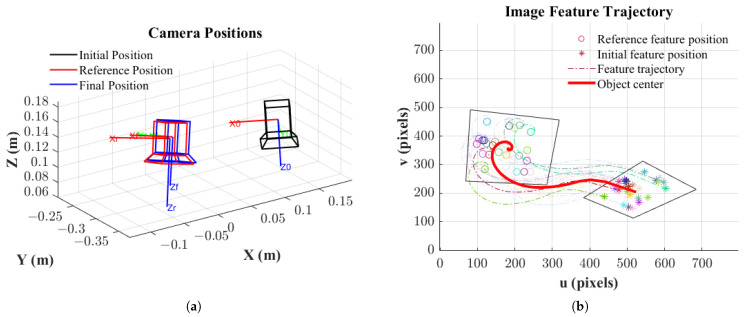
(**a**) Camera positions. (**b**) Feature trajectories.

**Figure 8:**
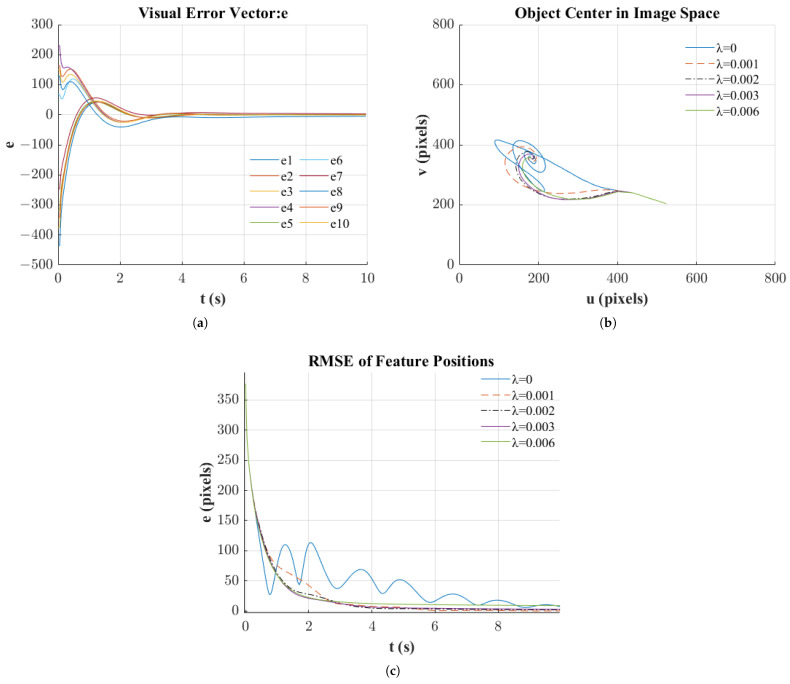
(**a**) Task error. (**b**) Object center. (**c**) RMSE.

The initial and final camera positions, as well as the camera’s target position, are shown in Figure 7a. It can be observed that the final camera position coincides with the reference position. Figure 7b displays the motion trajectories of 25 feature points, with “*” denoting their initial positions and hollow circles “O” indicating their target positions. Solid dots “.” represent the trajectories of the feature points, illustrating their gradual convergence towards the reference positions. Furthermore, in Figure 8a, the defined error vector e, as described in Equation (16), also converges to zero. This convergence indicates that as e converges, the image feature error also converges, and the camera’s pose aligns with the reference pose (R = I, t = 0).

λ is a critical parameter controlling the strength of geometric constraints in our model. As shown in Figure 8a,c, selecting an appropriate value of λ significantly influences both the convergence rate and stability of the neural network training process. Figure 8b illustrates the trajectory of feature point centers in image space, while Figure 8c demonstrates the evolution of feature point position errors over time.

When λ=0, the network exhibits oscillatory behavior with slower convergence, as evidenced by the fluctuations in Root Mean Square Error (RMSE) shown in Figure 8c. Introducing moderate constraint penalties (λ=0.001 to λ=0.003) effectively dampens these oscillations and accelerates convergence, leading to smoother trajectories in image space as depicted in Figure 8b. However, we observe that when λ becomes too large (λ=0.006), the system exhibits different limitations. While initial error reduction is rapid, when feature point errors become small, the convergence rate significantly decreases, and the system struggles to minimize the residual errors as shown in the latter part of Figure 8c. This trade-off suggests that intermediate values (around λ=0.003) provide the optimal balance between fast convergence, stability, and final accuracy.

#### 5.2.2. Comparisons with Other Studies

Currently, model-free visual servoing systems can be categorized into two approaches. The first category encompasses neural network-based methods [17,18,19,21], which typically use feature point positions as inputs/outputs, resulting in high-dimensional networks when processing numerous feature points. The second category consists of numerical iteration-based methods, such as Broyden’s method [15,16,20], which avoid explicit modeling by iteratively updating the Jacobian matrix. To validate the effectiveness of our proposed method, we conducted a series of simulations in MATLAB R2024a (MathWorks, Natick, MA, USA), comparing our geometry-constrained learning-based visual servoing framework with two representative model-free approaches: Broyden’s update method [20] and a neural network-based visual servoing method [21]. To ensure a fair comparison, all methods were independently tuned to achieve their best performance under the same experimental conditions. In addition, for the neural network-based method [21], we implemented a structure similar to ours, using a CMAC network with four overlapping associative memory layers. Each input dimension was discretized into nine regions, with every layer internally organizing the inputs into three blocks per dimension.

In the simulation environment, we constructed scenarios with 100 feature points and evaluated performance under both noise-free and noisy image conditions. The evaluation metrics included the RMSE of feature positions and the convergence trajectory of the object center in image space.

**Figure 9:**
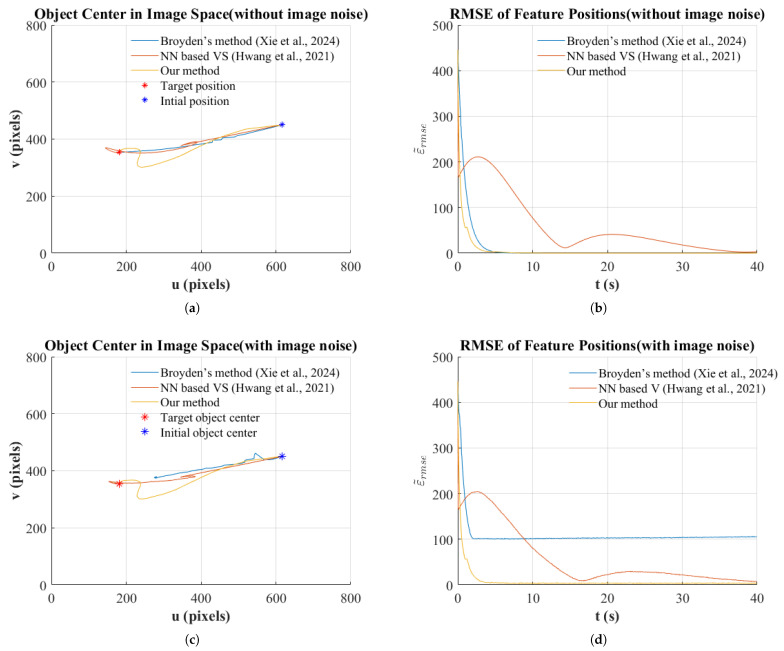
(**a**) Position of object center without image noise. (**b**) RMSE of feature positions without image noise. (**c**) Position of object center with image noise. (**d**) RMSE of feature positions with image noise [20,21].

As shown in Figure 9b, under noise-free conditions, all three methods eventually achieved convergence, but with significant differences in their convergence characteristics. Both our proposed method and Broyden’s method [20] demonstrated superior dynamic performance, rapidly reducing RMSE to near zero within approximately 6 s. Among these, our method achieved the fastest convergence rate. In contrast, the neural network-based method [21], while ultimately converging, exhibited notable fluctuations and required approximately 35 s to fully stabilize.

When image noise was introduced, the differences in robustness among the methods became more distinct. In this test, we applied random noise with a maximum amplitude of 20 pixels to 10 feature points to simulate measurement uncertainties in real-world environments. Under these conditions, our method maintained its rapid convergence characteristics and minimal steady-state error, while the comparative methods showed significant performance degradation as shown as Figure 9d. Specifically, the neural network-based method [21] eventually converged but required longer convergence time. Broyden’s method [20] demonstrated noticeable performance degradation, with its RMSE stabilizing at approximately 100 pixels, indicating high sensitivity to noise.

Figure 9a,c further visualize the object center trajectories in image space under noisy and noise-free conditions, respectively. The results clearly indicate that, in both scenarios, our method produces smoother trajectories and successfully converges to the target position even in the presence of noise.

Through these two simulation comparisons, we can conclude that our proposed algorithm outperforms existing methods in terms of convergence speed, stability, and noise robustness. This performance improvement can be attributed to two key innovations:

(1) Using a fixed-dimension task space error function as the neural network input effectively reduces network complexity. This smaller input dimension results in fewer learnable parameters, making the learning process more efficient. In contrast, other neural networks use feature point positions as inputs, where the input dimension increases proportionally with the number of feature points. As a result, when the number of feature points is large, these methods require learning an excessive number of parameters, significantly increasing computational complexity.
(2) Incorporating geometric constraints of feature points to assist network learning, which not only accelerates the learning process, but also ensures the physical feasibility of control outputs. In comparison, although Broyden’s method [20] avoids the problem of high-dimensional neural networks, its stability and convergence performance when processing image noise remain significantly inadequate, limiting its effectiveness in practical applications.
(3) Leveraging the Projective Homography matrix to enhance noise robustness. Unlike methods that rely on individual feature points, homography-based estimation effectively filters out the influence of measurement noise and ensures a more stable and reliable Jacobian estimation.

#### 5.2.3. Computational Complexity Analysis

To evaluate the computational efficiency of the proposed method, we compare it with two representative data-driven image-based visual servoing (IBVS) approaches: Broyden’s update method [20] and a fuzzy CMAC-based neural network method [21].

The method in [21] builds a fuzzy CMAC controller for each joint and uses the image feature error vector, which has a dimension of 2P, as input. As a result, both the complexity of the inference and the training process grow linearly with the number of joints $n_{q}$ and the number of feature points *P*, leading to a complexity per step of $O(n_{q}\cdot P)$.

The method in [20] updates the Jacobian matrix and a dynamic projection matrix during each iteration. Although it avoids direct matrix inversion, the update process still involves matrix multiplications and control computation. This results in a per-step complexity of $O(P\cdot n_{q}+n_{q}^{2})$, which increases significantly when *P* becomes large.

In contrast, the proposed method uses a compact input vector composed of homography-based task error and joint states, with a fixed dimension that does not depend on the number of feature points. The inference complexity per step is only $O(n_{q})$. This design reduces computational cost and improves real-time performance in visual tasks with many image features.

### 5.3. Experiments

**Figure 10:**
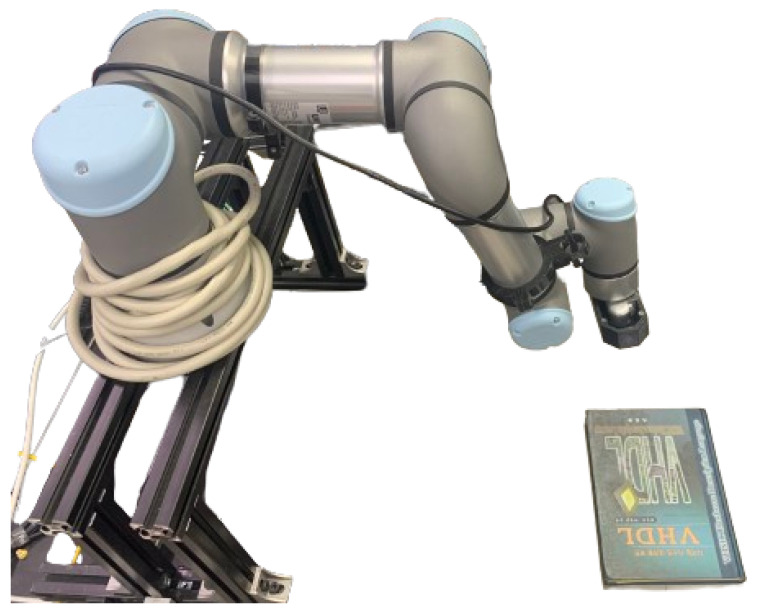
Receptive field organization.

As shown in Figure 10, the experimental setup consists of a 6-DOF UR5 collaborative robot with an Intel RealSense D435i RGB-D camera (30 FPS) rigidly mounted on its end-effector. The visual servoing system was implemented using the Robot Operating System (ROS). A visual processing node (20 Hz) performed feature extraction, matching, and control computation. A control interface node (125 Hz) converted the control outputs into joint velocity commands. Communication between nodes was handled via ROS topics over TCP/IP. Computation was performed on a workstation with an Intel i7-9700K CPU, 32 GB RAM, and an NVIDIA RTX 4080Ti GPU.

To validate the effectiveness of our algorithm, we conducted two comparative experiments using different geometric constraint parameters: λ. Both experiments tested robustness by introducing occlusions of partial feature points during robot motion.

In both experiments, the robot was initially programmed to follow a random trajectory near its starting position to collect pre-training data. Feature matching and homography estimation were performed using the LightGlue framework combined with RANSAC, ensuring robust performance under varying lighting conditions and viewpoint changes while effectively filtering out outliers.

**Figure 11:**
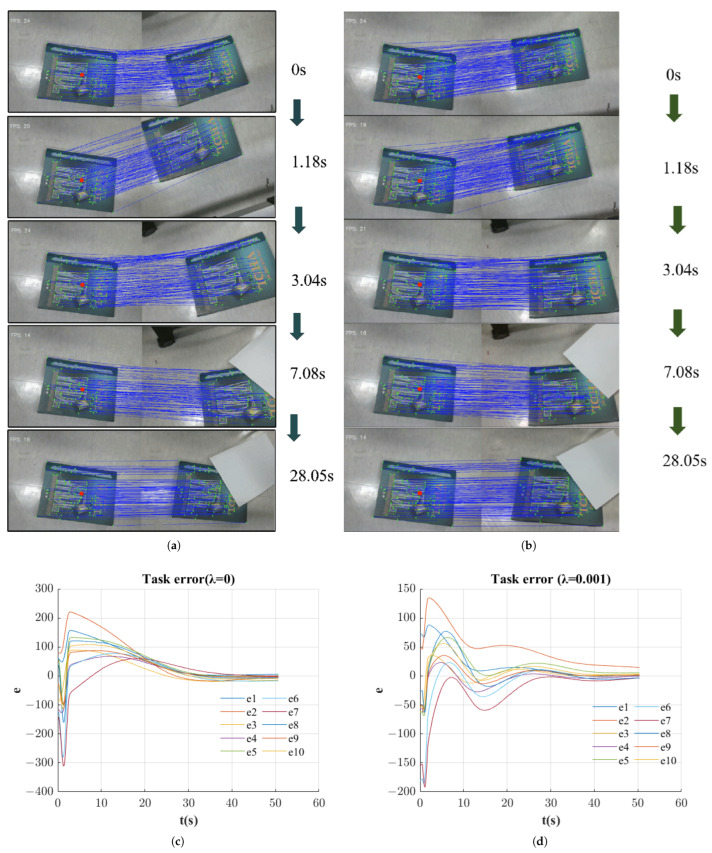
(**a**) Experiment 1.(**b**) Experiment 2. (**c**) Task error of experiment 1. (**d**) Task error of experiment 2.

In the first experiment (λ=0), Figure 11a shows the progress of the images captured during online learning compared to the reference images. The sequence displays images at the initial moment and at 1.18 s, 7.08 s, and 28.05 s, respectively. Figure 11c tracks the evolution of the task error e, revealing an initial error increase during the first 1 s due to insufficient neural network learning, followed by eventual convergence as learning progressed.

**Figure 12:**
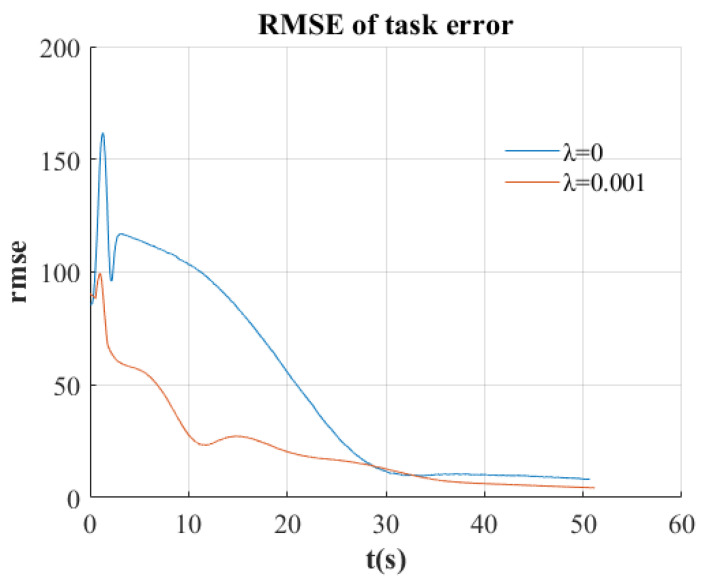
RMSE of feature positions for two experiments.

In the second experiment (λ=0.001), the system exhibited enhanced performance characteristics, as illustrated in Figure 11b,d. These figures demonstrate accelerated convergence toward the reference image compared to the unconstrained approach. Figure 12 presents the comparison of task error RMSE between both experimental conditions, revealing the advantages of incorporating geometric constraints. The constrained implementation (red line) achieves error reduction at a substantially higher rate.

Throughout both experiments, we deliberately occluded certain feature points at various times, yet the error consistently converged. This demonstrates our algorithm’s robustness in maintaining accuracy even under challenging conditions with varying numbers of visible feature points.

The experimental results confirm that incorporating geometric constraints through our proposed (λ) parameter significantly accelerates learning.

## 6. Conclusions

In this paper, we introduce a geometry-constrained learning-based controller for visual servoing systems based on projective homography. Our approach utilizes a neural network to learn the system’s Jacobian matrix, thereby eliminating the need for precise camera calibration parameters and detailed robot kinematic models. Unlike other neural network approaches, our method utilizes a newly defined error vector related to projective homography as input, ensuring a constant input size irrespective of the number of image feature points. We also demonstrated in Appendix A that the defined error vector e = 0 is a sufficient and necessary condition to achieve R = I and t = 0, which signifies the alignment of the camera with the target. Furthermore, we incorporated geometric constraints between feature points in the network update process. By ensuring that model predictions conform to the fundamental principles of projective geometry, we significantly improved learning efficiency. Through simulations and experiments, we validate that our approach achieves superior performance compared to other model-free visual servoing methods, exhibiting faster convergence rates, enhanced robustness to image noise and partial occlusions.

Looking forward, while the proposed framework is robust and calibration-free, it still assumes sufficient exploration and network convergence during deployment. In practice, especially in unfamiliar or dynamic environments, learning may be incomplete, potentially leading to unsafe control actions. As a future direction, we plan to embed safety constraints into the control law, such as limits on joint velocities, workspace boundaries, and proximity to humans or delicate objects. These constraint-aware mechanisms will help ensure safe robot behavior even under imperfect Jacobian estimation, improving system reliability and enabling deployment in safety-critical applications.

## Data Availability

Dataset available on request from the authors.

## Appendix A

Necessity: e=0 -> “R=I and t=0” If e=0, we have $E^{i}\left(\begin{array}{c} \varepsilon_{\mathrm{di}}\\ 1 \end{array}\right)=\varepsilon_{\mathrm{di}}-\varepsilon_{i}=0$. This implies that the feature points in the reference and current images are perfectly aligned. For a given set of four non-collinear points $\varepsilon_{\mathrm{ci}}$ and $\varepsilon_{\mathrm{di}}$(i=1,2,3,4,5), their transformation uniquely determines the projective homography matrix. Therefore, $\bar{G}=I$ and $\gamma_{i}=1$.

Because of $\bar{G}=\frac{G}{\det{\left(G\right)}^{1/3}}$, we can derive $G=\det{\left(G\right)}^{1/3}I$, which implies the G is a diagonal matrix with all elements on its diagonal being $\det{\left(G\right)}^{1/3}$. According to Equation (4), we obtain:

(A1) $$H=K^{-1}\mathrm{GK}=\det{\left(G\right)}^{1/3}I$$

H multiplies the unit vectors $i_{x}={\left[\begin{array}{ccc} 1 & 0 & 0 \end{array}\right]}^{T}$, $i_{y}={\left[\begin{array}{ccc} 0 & 1 & 0 \end{array}\right]}^{T}$ and $i_{z}={\left[\begin{array}{ccc} 0 & 0 & 1 \end{array}\right]}^{T}$, resulting in following equations:

(A2) $$Hi_{x}=Ri_{x}\;+tn_{d1}=\det{\left(G\right)}^{1/3}i_{x}$$

(A3) $$Hi_{y}=Ri_{y}\;+tn_{d2}=\det{\left(G\right)}^{1/3}i_{y},$$

(A4) $$Hi_{z}=Ri_{z}\;+tn_{d2}=\det{\left(G\right)}^{1/3}i_{z},$$

where $n_{di}$ represents i-th element of the vector $n_{d}$.

The expressions $\left(\mathrm{A2}\right)\cdot n_{d2}-\;\left(\mathrm{A3}\right)\cdot n_{d1}$, $\left(\mathrm{A3}\right)\cdot n_{d3}-\;\left(\mathrm{A4}\right)\cdot n_{d2}$ and $\left(\mathrm{A4}\right)\cdot n_{d1}-\;\left(\mathrm{A2}\right)\cdot n_{d3}$ can be simplified as:

(A5) $$Rv_{i}=\det{\left(G\right)}^{1/3}v_{i}$$

where $v_{1}=\left(i_{x}n_{d2}-i_{y}n_{d1}\right)$, $v_{2}=\left(i_{y}n_{d3}-i_{z}n_{d2}\right)$ and $v_{3}=\left(i_{z}n_{d1}-i_{x}n_{d3}\right)$.

Because R rotates the vector $v_{i}$ without changing its length, it ensures that the norm of the rotated vector $∥Rv_{i}∥$ is equal to the norm of the original vector $∥v_{i}∥$, implying:

(A6) $$\det{\left(G\right)}^{1/3}=1$$

We assume that $n_{d}\neq 0$. From of Equations (A5) and (A6), we can further conclude that:

(A7) R=I

Substituting Equations (A6) and (A7) into Equation (2) yields:

(A8) t=0

Sufficiency: “R=I and t=0” -> e=0.

It is evident that the desired camera frame $\left\{F_{cd}\right\}$ coincides with the current camera frame $\left\{F_{c}\right\}$ when R = I and t = 0. Consequently, the depth ratio $\frac{Z_{di}}{Z_{i}}$ equals 1, and $\gamma_{i}=1$. Additionally, we have $\bar{G}=\frac{1}{\det{\left(KK^{-1}\right)}^{1/3}}\left(KK^{-1}\right)=I$. Therefore, e=0 is proven in term of the Equations (13)–(16).

## References

[1] Thomas J., Chaumette F. (2024). Positioning in Congested Space by Combining Vision-based and Proximity-based Control. IEEE Robot. Autom. Lett..

[2] Leite G.R., Araújo Í.B.Q.d., Martins A.d.M. (2023). Regularized Maximum Correntropy Criterion Kalman Filter for Uncalibrated Visual Servoing in the Presence of Non-Gaussian Feature Tracking Noise. Sensors.

[3] Gans N.R., Hutchinson S.A. (2007). Stable visual servoing through hybrid switched-system control. IEEE Trans. Robot..

[4] AlBeladi A., Ripperger E., Hutchinson S., Krishnan G. (2022). Hybrid eye-in-hand/eye-to-hand image based visual servoing for soft continuum arms. IEEE Robot. Autom. Lett..

[5] García-Aracil N., Malis E., Aracil-Santonja R., Pérez-Vidal C. (2005). Continuous visual servoing despite the changes of visibility in image features. IEEE Trans. Robot..

[6] Fang Y., Dixon W.E., Dawson D.M., Chawda P. (2005). Homography-based visual servo regulation of mobile robots. IEEE Trans. Syst. Man, Cybern. Part B (Cybernetics).

[7] Hu G., MacKunis W., Gans N., Dixon W.E., Chen J., Behal A., Dawson D. (2009). Homography-based visual servo control with imperfect camera calibration. IEEE Trans. Autom. Control..

[8] Chen J., Dawson D.M., Dixon W.E., Behal A. (2005). Adaptive homography-based visual servo tracking for a fixed camera configuration with a camera-in-hand extension. IEEE Trans. Control. Syst. Technol..

[9] Lai B., Li Z., Li W., Yang C., Pan Y. (2023). Homography-based visual servoing of eye-in-hand robots with exact depth estimation. IEEE Trans. Ind. Electron..

[10] Lei X., Fu Z., Spyrakos-Papastavridis E., Pan J., Li M., Chen X. (2023). IHUVS: Infinite Homography-Based Uncalibrated Methodology for Robotic Visual Servoing. IEEE Trans. Ind. Electron..

[11] Gong Z., Tao B., Yang H., Yin Z., Ding H. (2017). An uncalibrated visual servo method based on projective homography. IEEE Trans. Autom. Sci. Eng..

[12] Liu C., Ye C., Shi H., Lin W. (2024). Discrete-Time Visual Servoing Control with Adaptive Image Feature Prediction Based on Manipulator Dynamics. Sensors.

[13] Aghili F. (2021). Fault-tolerant and adaptive visual servoing for capturing moving objects. IEEE/ASME Trans. Mechatron..

[14] Xu F., Zhang Y., Sun J., Wang H. (2022). Adaptive visual servoing shape control of a soft robot manipulator using bezier curve features. IEEE/ASME Trans. Mechatron..

[15] Piepmeier J.A., McMurray G.V., Lipkin H. A dynamic quasi-Newton method for uncalibrated visual servoing. Proceedings of the 1999 IEEE International Conference on Robotics and Automation (cat. no. 99CH36288C).

[16] Piepmeier J.A., Lipkin H. (2003). Uncalibrated eye-in-hand visual servoing. Int. J. Robot. Res..

[17] Tokuda F., Arai S., Kosuge K. (2021). Convolutional neural network-based visual servoing for eye-to-hand manipulator. IEEE Access.

[18] Gao J., Proctor A., Bradley C. (2015). Adaptive neural network visual servo control for dynamic positioning of underwater vehicles. Neurocomputing.

[19] Tan N., Yu P., Zheng W. (2022). Uncalibrated and unmodeled image-based visual servoing of robot manipulators using zeroing neural networks. IEEE Trans. Cybern..

[20] Xie Z., Zheng Y., Jin L. (2024). A data-driven image-based visual servoing scheme for redundant manipulators with unknown structure and singularity solution. IEEE Trans. Syst. Man, Cybern. Syst..

[21] Hwang M., Chen Y.J., Ju M.Y., Jiang W.C. (2021). A fuzzy CMAC learning approach to image based visual servoing system. Inf. Sci..

[22] Al-Shanoon A., Lang H. (2022). Robotic manipulation based on 3-D visual servoing and deep neural networks. Robot. Auton. Syst..

[23] Hay O.A., Chehadeh M., Ayyad A., Wahbah M., Humais M.A., Boiko I., Seneviratne L., Zweiri Y. (2023). Noise-tolerant identification and tuning approach using deep neural networks for visual servoing applications. IEEE Trans. Robot..

[24] DeTone D., Malisiewicz T., Rabinovich A. Superpoint: Self-supervised interest point detection and description. Proceedings of the IEEE Conference on Computer Vision and Pattern Recognition Workshops.

[25] Lindenberger P., Sarlin P.E., Pollefeys M. Lightglue: Local feature matching at light speed. Proceedings of the IEEE/CVF International Conference on Computer Vision.

